# Hernie diaphragmatique étranglée: un piège à ne pas méconnaître

**DOI:** 10.11604/pamj.2015.21.27.6688

**Published:** 2015-05-13

**Authors:** Ammar Mahmoudi, Randa Salem

**Affiliations:** 1 Service de Chirurgie Générale et Digestive, CHU Fattouma Bourguiba de Monastir, Monastir, Tunisie; 2 Service d'Imagerie Médicale, CHU Fattouma Bourguiba de Monastir, Monastir, Tunisie

**Keywords:** Plaie thoracique, hernie diaphragmatique, occlusion, tomodensitométrie, chirurgie, chest wound, diaphragmatic hernia, occlusion, CT scan, surgery

## Image en medicine

La hernie diaphragmatique post-traumatique constitue une lésion particulière en traumatologie car elle risque de passer inaperçue et peut représenter ainsi un piège diagnostique. Le diagnostic se fait alors à l'occasion d'une complication, en particulier l'étranglement. La coupole diaphragmatique gauche est la plus fréquemment touchée. Nous rapportons le cas d'un patient âgé de 28 ans qui a été victime d'une plaie par arme blanche basithoracique gauche il y a trois ans et la radiographie du thorax avait montré un hémothorax gauche minime ayant bien évolué sous simple surveillance. Le patient avait consulté pour un syndrome occlusif sans signe respiratoire. La tomodensitométrie avait objectivé une hernie diaphragmatique gauche étranglée contenant le colon transverse gauche et l'épiploon comblant ainsi la cavité pleurale homolatérale jusqu'à l'apex pulmonaire refoulant légèrement le médiastin et le poumon avec une atélectasie passive en regard. Cet étranglement était responsable d'une distension majeur du colon d'amont et des dernières anses iléales. Le patient était opéré en urgence par laparotomie ayant permis la réintégration du colon (qui était souffrant mais qui a récupéré) dans la cavité péritonéale, la résection de l'épiploon de mauvaise qualité et une réparation du défect diaphragmatique par raphie simple. L'évolution était favorable avec une sortie à J5 et un suivi sans particularités. L'éventualité de la survenue d'une hernie diaphragmatique doit être systématiquement présente à l'esprit en cas de traumatisme thoraco-abdominal fermé violent ou en cas de plaie basithoracique. Il est nécessaire de continuer la surveillance et de réaliser, au moindre doute, une exploration par une tomodensitométrie.

**Figure 1:**
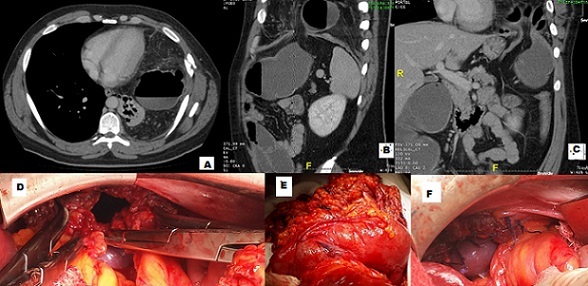
(A) tomodensitométrie thoraco-abdominale en coupe axiale montrant une image hydroaérique colique en intrathoracique gauche associée à une infiltration de la graisse péricolique, ainsi que l'épiploon comblant la cavité pleurale homolatérale; (B) tomodensitométrie thoraco-abdominale avec reconstruction frontale montrant une interruption de la coupole diaphragmatique gauche (le collet), avec présence du côlon transverse gauche en intrathoracique, une distension du colon en amont et des dernières anses iléales; (C) tomodensitométrie thoraco-abdominale avec reconstruction frontale montrant l'étranglement colique transverse gauche à travers la hernie diaphragmatique gauche; (D) vue opératoire montrant le défect diaphragmatique (le collet de la hernie) légèrement élargi pour pouvoir réintégrer le colon et l'épiploon dans l'abdomen; (E) vue opératoire montrant le contenu de la hernie diaphragmatique après sa réduction dans l'abdomen: colon transverse gauche très distendue avec la zone de striction et l'épiploon en majeur partie souffrant; (F) vue opératoire montrant la suture du défect diaphragmatique gauche

